# Discovery of a Novel Long Noncoding RNA *Lx8-SINE B2* as a Marker of Pluripotency

**DOI:** 10.1155/2021/6657597

**Published:** 2021-02-06

**Authors:** Fuquan Chen, Miao Zhang, Xiao Feng, Xiaomin Li, Haotian Sun, Xinyi Lu

**Affiliations:** State Key Laboratory of Medicinal Chemical Biology, College of Pharmacy, Nankai University, Tianjin 300307, China

## Abstract

Pluripotency and self-renewal of embryonic stem cells (ESCs) are marked by core transcription regulators such as Oct4, Sox2, and Nanog. Another important marker of pluripotency is the long noncoding RNA (lncRNA). Here, we ind that a novel long noncoding RNA (lncRNA) *Lx8-SINE B2* is a marker of pluripotency. LncRNA *Lx8-SINE B2* is enriched in ESCs and downregulated during ESC differentiation. By rapid amplification of cDNA ends, we identified the full-length sequence of lncRNA *Lx8-SINE B2*. We further showed that transposable elements at upstream of lncRNA *Lx8-SINE B2* could drive the expression of lncRNA *Lx8-SINE B2*. Furthermore, ESC-specific expression of lncRNA *Lx8-SINE B2* was driven by Oct4 and Sox2. In summary, we identified a novel marker lncRNA of ESCs, which is driven by core pluripotency regulators.

## 1. Introduction

Most of the mammalian genome is composed of noncoding sequences. Among them, transposable elements (TEs) contribute to ~40% of the genome [1]. The majority of TEs are silenced, however, a small percentage of TEs are expressed during development and in diseases [2]. They play multiple roles in these processes, including function as enhancers, promoters, and long noncoding RNAs (lncRNAs) [3–6]. In vertebrates, 70% lncRNAs are composed of TEs [7]. TEs also confer tissue-specific expression on lncRNAs through the recruitment of transcription factors [3, 4, 6]. TE-derived lncRNAs actively participate in development. TE-derived lncRNA ROR functions as a sponge to miRNA and also works with hnRNPA1 to promote c-Myc expression during reprogramming [8–10]. Endogenous retrovirus HERVH-derived lncRNAs maintain pluripotency of human embryonic stem cells [3, 11–13]. Asymmetrical expression of ERV1 and ERVK-derived lncRNA LincGET in two- to four-cell mouse embryos biases cell fate toward inner cell mass [14]. These findings all suggest an important role of TE-derived lncRNA in development. Most of these findings are based on human cell lines. We are still lack of understanding of TE-derived lncRNAs in mouse embryonic stem cells (ESCs). In this study, we investigated the expression and regulation of one representative lncRNA *Lx8-SINE B2* in ESCs.

## 2. Methods

### 2.1. Cell Culture

Mouse ESCs (E14) were cultured on plates coated with 0.2% gelatin (G1890, Sigma) in medium with 15% fetal bovine serum (FBS, SH30070.03, Hyclone), 2 mM L-glutamine (Gibco), 1% penicillin-streptomycin (P1400, Solarbio), 0.1 mM nonessential amino acids (Gibco), 0.1 mM *β*-mercaptoethanol (M3148–250, Sigma), and 10 ng/ml leukemia inhibitory factor (LIF; Z03077, GenScript). Mouse embryonic fibroblasts (MEFs) and 3 -T3 cells were maintained on plates (703001, NEST Biotechnology) in Dulbecco's modified Eagle's medium (DMEM, Hyclone) supplemented with 10% FBS, 2 mM L-glutamine, and 1% penicillin-streptomycin. Cells were cultured at 37°C in CO_2_ incubator.

2i culture medium contain 50% DMEM/F12 (BasalMedia), 50% Neurobasal media (Gibco), 1% N2 supplement, 1% B27 (Gibco), 0.1 mM nonessential amino acids (Gibco), 2 mM L-glutamine(Gibco), 1% penicillin-streptomycin (P1400, Solarbio), 0.1 mM *β*-mercaptoethanol (M3148–250, Sigma), 1 *μ*M MEK inhibitor PD0325901 (T6189, TargetMol), and 3 *μ*M GSK3 inhibitor CHIR99021 (2520691, BioGems). 10 ng/ml leukemia inhibitory factor (LIF; Z03077, GenScript) was added for 2i/LIF condition.

### 2.2. RNA Extraction, Reverse Transcription, and Quantitative PCR (qPCR)

Total RNA was extracted with RNAiso Reagent (B9109, Takara) as described [15] and treated with DNase I to remove genomic DNA in DEPC water (B501005, Sangon Biotech). The cDNA synthesis was carried out in RNase-free tubes (401001, NEST Biotechnology) with the Transcriptor First Strand cDNA Synthesis Kit (4897030001, Roche), according to the manufacturer's instructions. Quantitative PCR (qPCR) reactions were performed using the Hieff qPCR SYBR Green Master Mix (H97410, Yeasen) in a QuantStudio 6 Real-Time PCR System (Life Technologies). Primer sequences for qPCR analysis are listed in Table 1.

### 2.3. Depletion of Gene Expression with shRNAs

For gene knockdown, short hairpin RNAs (shRNAs) for luciferase (control) or target genes were designed by an online tool (http://sirna.wi.mit.edu/) and synthesized by GENEWIZ corporation. The shRNA plasmids were constructed using the pSuper-puro system and purified with a kit (1211-01, Biomiga). mESCs were transfected with DNA using Polyjet (SL100688, SignaGen), according to the manufacturer's protocol. Transfected ESCs were selected with 1 *μ*g/ml puromycin from 24 h after transfection. After four days of puromycin selection, transfected cells were harvested. The sequences of shRNAs are listed in Table 2.

### 2.4. 5′ and 3′ Rapid Amplification of cDNA Ends (RACE) Analysis

For 3′ RACE, first-strand cDNA synthesis is initiated at the poly(A) tail of total RNA using the anneal oligo(dT)-containing RT Adapter Primer (AP) to mRNA. Gene-specific primer pF1 was designed based on the known sequence. 3′ fragment was amplified by primer pF1 and general primer gR1, the RACE PCR products were separated on a 1.5% agarose gel.

For 5′ RACE, the first-strand cDNA was synthesized from total RNA using a gene-specific primer (RT GSP1), which was designed according to the 3′ known sequence. A homopolymer tail was subsequently added to the 3′-end of the cDNA using terminal deoxynucleotidyl transferase kit (2230A, Takara), according to the manufacturer's instruction. First-round PCR was performed based on poly(C) tail designed dG adaptor primer to synthesize double-stranded cDNA. Then, general primer gP1 and gene-specific primer pR2 were used for second-round PCR to amplify the cDNA 5′ end sequence. The RACE PCR products were separated on a 1.5% agarose gel and cloned into pEASY-T1 (TransGen Biotech) for Sanger sequencing. The gene-specific RACE primers used for mapping each end were from Sangon Biotech and were listed in Table 3.

### 2.5. Dual-Luciferase Reporter Gene Assay

Mouse ESCs were seeded at a density of 8 × 10^4^ cells per well in a 24-well plate. Luciferase assay was performed as previously described [16]. The total amount of 200 ng of the various promoters of lncRNA *Lx8-SINE B2* or pGL4.23 empty vector was transfected into each well of E14 ESC on a 24-well plate together with 10 ng of pCMV-Renilla. The medium was changed 12 h after transfection. After transfection of 36 h, cells were collected and lysed in 1x passive lysis buffer. The luciferase activity was determined by Dual-Luciferase Reporter Assay System (#E1910, Promega) according to the manufacturer's instructions.

### 2.6. Statistical Analysis

Data were analyzed with Student's *t*-test (two-tailed). Significant differences were defined as ns for nonsignificant, ^∗∗^*p* < 0.01, and ^∗∗∗^*p* < 0.001.

## 3. Results

### 3.1. Mapping the Full-Length Sequence of lncRNA *Lx8-SINE B2*

**Figure 1:**
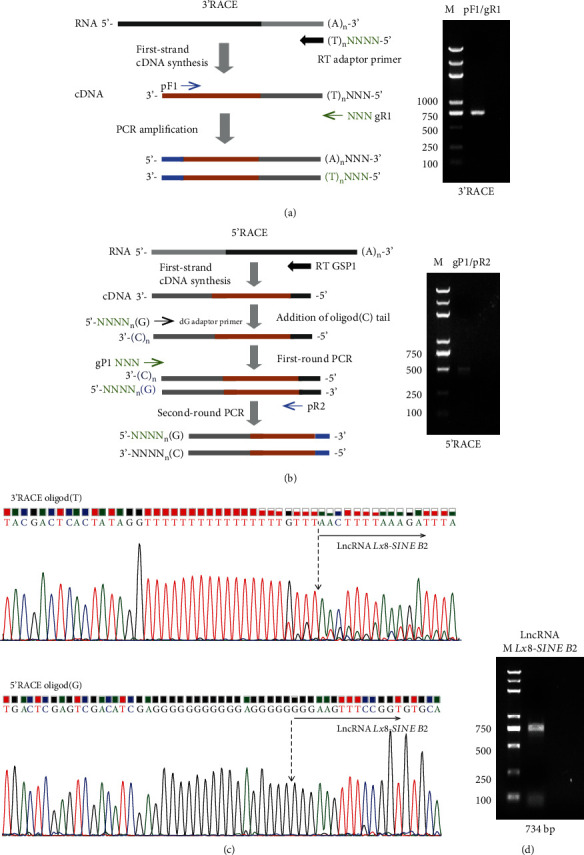
Mapping the full-length sequence of lncRNA *Lx8-SINE B2*. (a) Schematic of the 3′-rapid amplification of cDNA ends (RACE) (left) and 3′ RACE result for lncRNA *Lx8-SINE B2* (right). (b) Schematic of the 5′ RACE and its result for lncRNA *Lx8-SINE B2*. (c) DNA sequencing of RACE using a universal primer in pEASY-T1 vector. (d) Validation of lncRNA *Lx8-SINE B2* transcript size by PCR from cDNA. M, DNA marker.

Through mining the previous publication [17], it was shown that lincRNA-1282 was expressed in ESCs and its depletion leads to downregulation of c-Myc [17], which is an important reprogramming factor. Therefore, we set out to perform RACE to identify the full-length of lincRNA-1282 [17], which is a partial sequence of lncRNA *Lx8-SINE B2*. To identify the full length of *Lx8-SINE B2*, we performed 3′ RACE and 5′ RACE with primers as designed (Figures 1(a) and 1(b)). Our amplicons for both 5′ and 3′ RACE were visible as a single DNA band without multiple or unspecific bands (Figures 1(a) and 1(b)). Next, we sequenced the amplicons and identified the sequences of lncRNA *Lx8-SINE B2* (Figure 1(c)). With the 5′ and 3′ ends of lncRNA *Lx8-SINE B2* found, we designed primers to amplify the full length of lncRNA *Lx8-SINE B2* and subcloned the lncRNA into TA cloning vector (Figure 1(d)). The lncRNA *Lx8-SINE B2* was revealed to be a 734 bp lncRNA.

### 3.2. Expression Pattern of lncRNA *Lx8-SINE B2*

**Figure 2:**
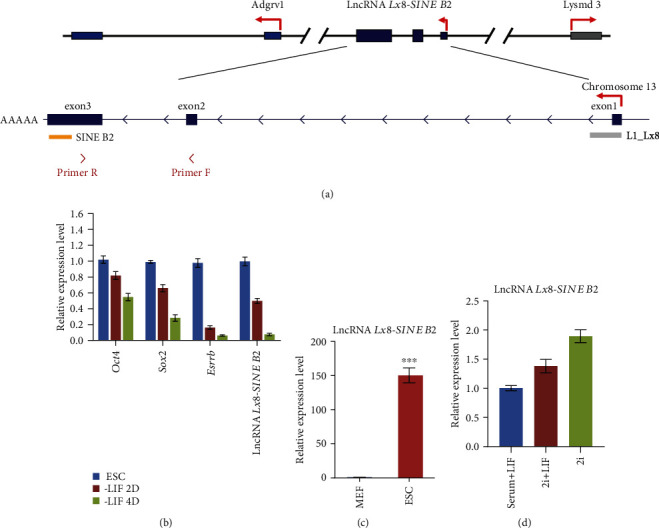
Genomic location and expression of lncRNA *Lx8-SINE B2*. (a) Schematic of the mouse lncRNA *Lx8-SINE B2* locus on chromosome 13. lncRNA *Lx8-SINE B2* is between *Adgrv1* and *Lysmd3*. There are three exons and some retrotransposon fragments of LINE or SINE in lncRNA *Lx8-SINE B2*. RT–qPCR primers were indicated below. (b) The expression level of lncRNA *Lx8-SINE B2*, *Oct4 Sox2*, and *Esrrb* in mESCs in the presence or absence of LIF for 2-4 days, as measured by RT–qPCR and normalized to *Gapdh* levels. Biological-triplicate data (*n* = 3 dishes) are presented as mean ± s.e.m. (c) qPCR analysis of the expression level of lncRNA *Lx8-SINE B2* in mouse ESCs and MEF cells. ^∗∗∗^*p* < 0.001 according to two-sided Student's *t*-test (d) Expression analysis of lncRNA *Lx8-SINE B2* in ESCs cultured under serum/LIF, 2i/LIF or 2i condition. Biological-triplicate data (*n* = 3 extracts) are presented as mean ± s.e.m.

We searched the sequences of lncRNA *Lx8-SINE B2* against the mouse genome (mm10) and discovered that lncRNA *Lx8-SINE B2* contained 3 exons, which are located between *Adgrv1* and *Lysmd3* gene (Figure 2(a)). Exon 1 of lncRNA *Lx8-SINE B2* overlapped with LINE1 family Lx8 and its third exon overlapped with SINE B2 (Figure 2(a)); therefore, we named this lncRNA as *Lx8-SINE B2*. We designed primers on the nonrepeat region of exon 2 and 3 to detect the expression of lncRNA *Lx8-SINE B2*. Interestingly, it is noticed that lncRNA *Lx8-SINE B2*was downregulated during ESC differentiation, similar to the pluripotency gene *Oct4*, *Sox2*, and *Esrrb*, according to qPCR results (Figure 2(b)). We also found that lncRNA *Lx8-SINE B2* was also expressed in ESCs instead of differentiated cells such as MEF (Figure 2(c)). Furthermore, we demonstrated that the expression of lncRNA *Lx8-SINE B2*was not affected by the alternation of ESC culture condition. Its expression was slightly upregulated in the presence of 2i/LIF or 2i condition in contrast to the serum/LIF culture condition (Figure 2(d)). These suggest lncRNA *Lx8-SINE B2* as a marker of ESC.

### 3.3. Promoter Structure of lncRNA *Lx8-SINE B2*

**Figure 3:**
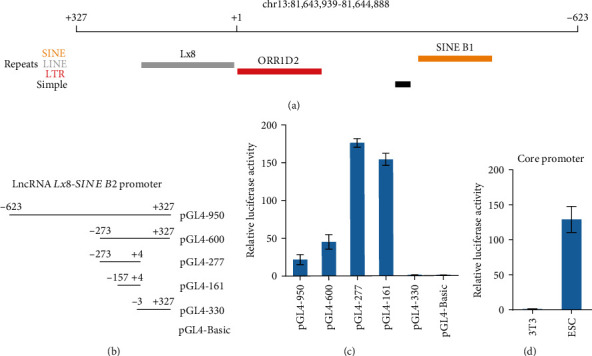
Dissection of lncRNA *Lx8-SINE B2* promoter region. (a) Schematic of the positions of TEs on according to mouse mm10 genome the promoter region of lncRNA *Lx8-SINE B2*. (b) Schematic of various length fragments of lncRNA *Lx8-SINE B2* promoter constructs. (c) Activities of various length fragments of lncRNA *Lx8-SINE B2* promoter constructs were determined by *luciferase* reporter gene assays in E14 ESCs. Biological-triplicate data (*n* = 3 dishes) are presented as mean ± s.e.m. (d) Luciferase assay analysis of core promoter activity of lncRNA *Lx8-SINE B2* in ESCs and 3T3 cells. Biological-triplicate data (n =3 extracts) are presented as mean ± s.e.m.

After that, we examined how the specific expression of lncRNA *Lx8-SINE B2* was achieved. The upstream 1 kb promoter region of lncRNA *Lx8-SINE B2* contains ORR1D2 and SINE B1 (Figure 3(a)). To study how *Lx8-SINE B2* is regulated in ESCs, we cloned -623 bp to +327 bp of lncRNA *Lx8-SINE B2* gene into luciferase reporter (Figures 3(a) and 3(b)). We also created various truncation versions of this region to identify the core promoter of *Lx8-SINE B2* (Figure 3(b)). The region corresponding to ERV, origin-region repeat 1 type D2 (ORR1D2, -157 bp to +3 bp) carried the strongest promoter activity in contrast to those of other truncations (Figure 3(c)). The promoter activity of ORR1D2 was specific to ESCs but inactivated in 3T3 fibroblasts (Figure 3(d)). These results support that lncRNA *Lx8-SINE B2* is driven by ERV ORR1D2, implicating that TEs not only contribute to the exons of lncRNAs but also the promoter of lncRNAs.

### 3.4. Transcriptional Regulation of lncRNA *Lx8-SINE B2* by Oct4 and Sox2

**Figure 4:**
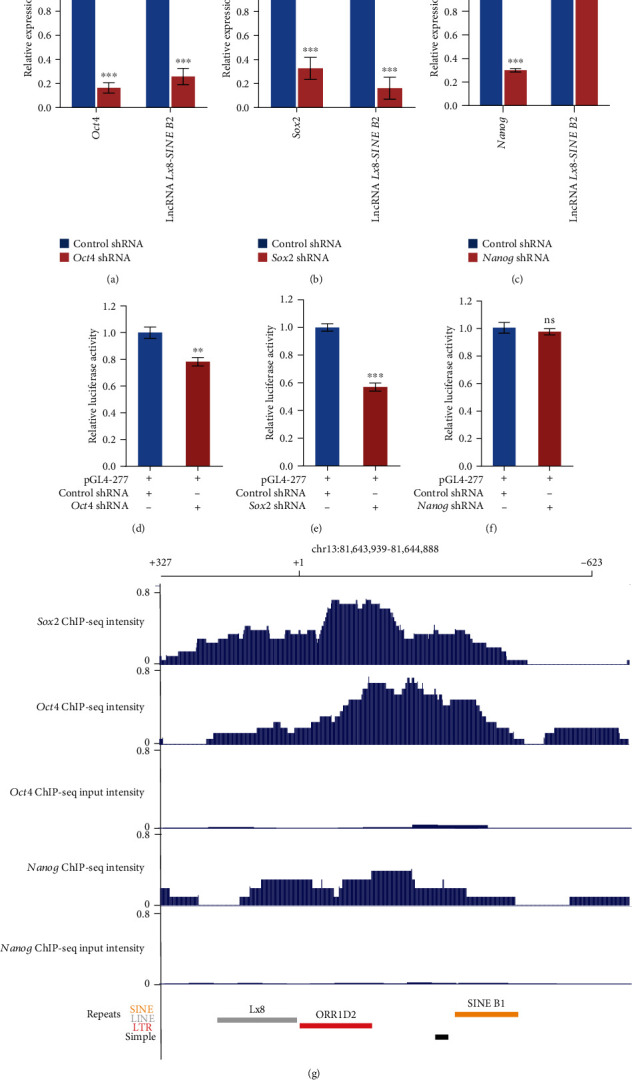
Oct4 and Sox2 drive the expression of lncRNA *Lx8-SINE B2*. (a–c) qPCR analysis of lncRNA *Lx8-SINE B2* expression after depletion of core transcription factors *Oct4* (a), *Sox2* (b), and *Nanog* (c) in ESCs. The data are represented as mean ± s.e.m. from three biological replicates. ns, non-significant; ^∗∗^*p* < 0.01; ^∗∗∗^*p* < 0.001 according to two-sided Student's *t*-test. Biological-triplicate data (*n* = 3 dishes). (d–f) Luciferase assay analysis of core promoter activity of lncRNA *Lx8-SINE B2* after depletion of core transcription factors *Oct4* (d), *Sox2* (e), and *Nanog* (f) in ESCs. Biological-triplicate data (*n* = 3 extracts) are presented as mean ± s.e.m. (g) Binding profile of Sox2, Oct4, and Nanog on the promoter region of lncRNA *Lx8-SINE B2* according to published data as described in methods. Input was included as a control.

To identify which transcription factor activates lncRNA *Lx8-SINE B2*, we depleted three core pluripotency transcription factors (Oct4, Sox2, and Nanog) (Figures 4(a)–4(c)). Depletion of *Oct4* or *Sox2*, but not *Nanog*, strongly suppressed lncRNA *Lx8-SINE B2* expression (Figures 4(a)–4(c)). We also examined the expression of lncRNA *Lx8-SINE B2* after the depletion of *Oct4*, *Sox2*, and *Nanog* (Figures 4(a)–4(c)). However, depletion of either *Sox2* or *Oct4*, but not *Nanog*, affected the promoter activity of ORR1D2 (Figures 4(d)–4(f)). *Sox2* depletion imposed stronger inhibition on ORR1D2 than Oct4 and Nanog (Figures 4(d)–4(f)). Furthermore, we examined the binding of Oct4, Sox2, and Nanog on the promoter of lncRNA *Lx8-SINE B2*. Consistent with results from luciferase assay, only Oct4 and Sox2 bound to the promoter according to our analysis of published ChIP-seq data (Figure 4(g)). These results suggest that Sox2 and Oct4 directly bind to ORR1D2 to activate *Lx8-SINE B2* in ESCs (Figure 4(g)).

**Figure 5:**
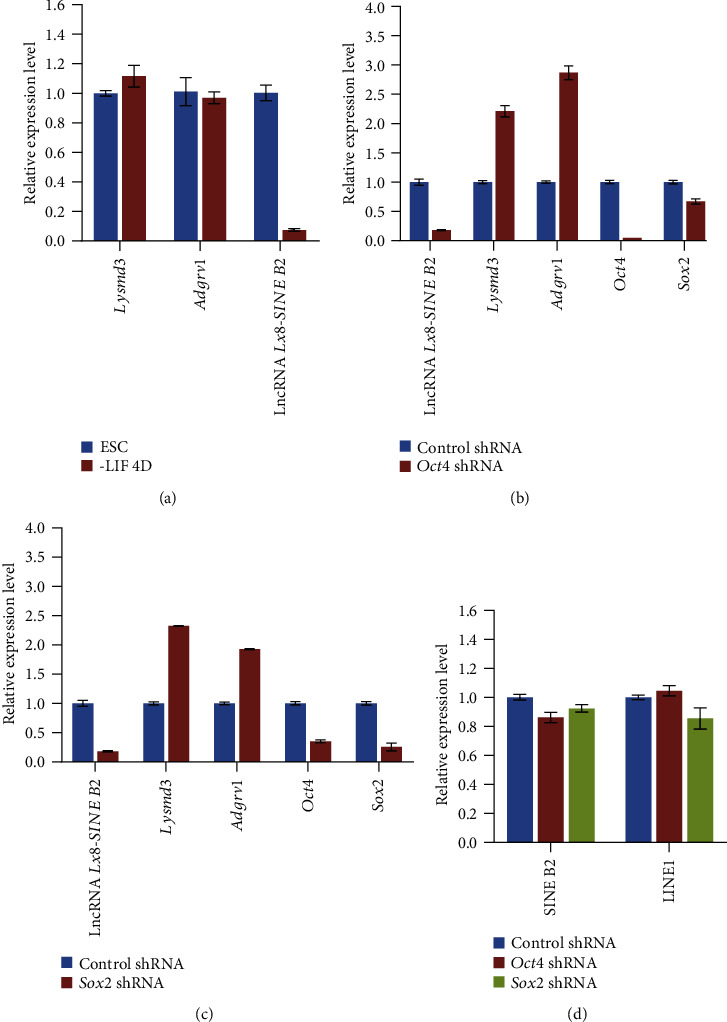
Expression of neighboring genes of *Lx8-SINE B2*. (a) qPCR analysis of neighboring genes (*Adgrv1* and *Lysmd3*) of lncRNA *Lx8-SINE B2* in ESCs cultured with or without LIF; (b, c) RT-qPCR analysis of lncRNA *Lx8-SINE B2*, *Adgrv1*, *Lysmd3* and pluripotent genes (*Oct4* and *Sox2*) expression after depletion of *Oct4* (b) or *Sox2* (c) in E14 ESCs. The data are represented as mean ± s.e.m. from three biological replicates. (d) Expression of LINE1 and SINE B2 after depletion of *Oct4* or *Sox2*.

To exclude the possibility that Oct4 and Sox2 activate neighboring genes of lncRNA *Lx8-SINE B2* together with it, we examined the expression of *Lysmd3* and *Adgrv1* during ESC differentiation. Different from lncRNA *Lx8-SINE B2*, both *Lysmd3* and *Adgrv1* were unaffected by LIF withdrawal (Figure 5(a)). Furthermore, the expression of *Lysmd3* and *Adgrv1* were activated by depletion of *Oct4* or *Sox2*, suggesting they are regulated differently from *Lx8-SINE B2* (Figures 5(b) and 5(c)). Moreover, the expression of LINE1 and SINE B2 were not affected by *Oct4* or *Sox2* depletion (Figure 5(d)), confirming the specificity of Oct4 and Sox2 in activating the expression of lncRNA *Lx8-SINE B2*.

## 4. Discussion

In summary, we identified a novel pluripotency marker lncRNA *Lx8-SINE B2*, whose expression is driven by the binding of Oct4 and Sox2 on ORR1D2. Oct4 and Sox2 are the core pluripotency regulators in ESCs [18, 19]. Oct4 and Sox2 can drive the expression of lncRNAs in cancer cells and ESCs [20–22]. The binding profiles of OCT4 are different in human and mouse ESCs [23], which can be explained by its binding differences on species-specific TEs [23]. Here, we found that Oct4 and Sox2 targeted mouse TE ORR1D2 to drive ESC-specific lncRNA expression (Figure 4), further supporting the important role of TEs in driving the expression of species-specific lncRNAs. There are many pluripotency markers; however, we provide *Lx8-SINE B2* as an additional novel marker of pluripotency. It lies at the downstream of key pluripotency genes *Oct4* and *Sox2* (Figure 4). It composes of TEs and is distinct from traditional markers of pluripotency. In comparison to other ESC markers, *Lx8-SINE B2* is unique as an ORR1D2-driven pluripotency marker, which demonstrates that transposable elements can function as cell type-specific lncRNA and promoter, similarly to protein-coding genes. Finally, its depletion is associated with the downregulation of *Myc* in ESCs [17]; therefore, *Lx8-SINE B2* expression also reflects *Myc* expression status of ESCs. Myc represses primitive endoderm differentiation [24]. Myc also maintains ESC pluripotency and self-renewal [25]. Therefore, we speculate that the depletion of lncRNA *Lx8-SINE B2* may cause a phenotype similar to that of Myc downregulation.

Our study demonstrates that different types of TEs combine to form lncRNA and drive lncRNA expression (Figures 2 and 3), implicating TEs as important components of lncRNA. TEs in lncRNAs work as an important RNA domain [26, 27]. TEs within lncRNAs regulate the tissue-specific expression of lncRNAs [4, 28]. In human, lncRNAs containing HERVH are specifically expressed in human ESCs [3, 4, 7]. TEs within lncRNAs also contribute to their functions. For example, SINE B2 in antisense lncRNA of *Uchl1* interacts with *Uchl1* mRNA and promotes the translation of Uchl1 through enhancing the association of mRNA with polysome [29]. These studies demonstrate that TEs are critical to the expression and function of lncRNAs. Given that lncRNA *Lx8-SINE B2* is composed of TE Lx8 and SINE B2, it will be interesting to investigate whether ORR1D2 drive the expression of other lncRNAs and the function of Lx8 and SINE B2 within lncRNAs in the future study.

## 5. Conclusion

In conclusion, we mapped the full-length sequence of lncRNA *Lx8-SINE B2* and found it as an ESC-specific lncRNA. We also found that it was driven by ORR1D2 which was bound by Sox2 and Oct4 to drive its transcription. These findings support TEs as important compositions and promoter of lncRNA.

## Figures and Tables

**Table 1:** Primer sequences for qPCR analysis.

Gene	Forward	Reverse
*lncRNA Lx8-SINE B2*	GCTGTTATGACTTGTTTCCTGGT	CTCTTCCTTGCAGGCTTAGAAC
*Oct4*	GTGGAAAGCAACTCAGAGG	GGTTCCACCTTCTCCAACT
*Sox2*	GCGGAGTGGAAACTTTTGTCC	CGGGAAGCGTGTACTTATCCTT
*Nanog*	TTGCTTACAAGGGTCTGCTACT	ACTGGTAGAAGAATCAGGGCT
*Esrrb*	GCACCTGGGCTCTAGTTGC	TACAGTCCTCGTAGCTCTTGC
*Prdm14*	CTCTTGATGCTTTTCGGATGACT	GTGACAATTTGTACCAGGGCA
*Lysmd3*	ACGGTTTCCCTCCCAGGAAT	CATCAAGTCTATCTCTCGATGCG
*Adgrv1*	CAGCCCTGAATCACTCTTCGT	CCCATCCAGGTCCGAGTCTA
LINE1	GGACCAGAAAAGAAATTCCTCCCG	CTCTTCTGGCTTTCATAGTCTCTGG
SINE B2	GAGTAAGAGCACCCGACTGC	AGAAGAGGGAGTCAGATCTCGT

**Table 2:** Targeting sequences of shRNAs.

Gene	shRNA target sequence
*Oct4* shRNA	GTGGAAAGCAACTCAGAGG
*Sox2* shRNA	GCGGAGTGGAAACTTTTGTCC
*Nanog* shRNA	TTGCTTACAAGGGTCTGCTACT

**Table 3:** RACE primers.

RACE primer name	Sequence
RT-adaptor primer	GCGAGCACAGAATTAATACGACTCACTATAGG(T)18VN
gR1	GCGAGCACAGAATTAATACGAC
pF1	ATACCTTCCTAAAACTAATGTGGACT
RT GSP1	TGAAGAACTTTTAGCACAGCAGC
dG-adaptor primer	GACTCGAGTCGACATCGAGGGGGGGGGGGGGGGGG
gP1	GACTCGAGTCGACATCG
pR2	CAACTGTTCTAAACGCTTCTTAG

## Data Availability

Published ChIP data analyzed by Cistrome [30] in this study are GSE54103 for Sox2 [31], GSE78073 for Oct4 [32], and GSE56312 for Nanog [33].
